# Supplementary material for: Vulnerability of the North Water ecosystem to climate change
Source: Nat Commun. 2021 Jul 22;12:4475. doi: 10.1038/s41467-021-24742-0 (PMC8298575; doi:10.1038/s41467-021-24742-0)
Supplement: Supplementary file 1 — Supplementary Info [file 41467_2021_24742_MOESM1_ESM.pdf]

Vulnerability of the North Water ecosystem to climate change

Supplementary Information

Supplementary Table 1 - Radiocarbon dates

Marine core (AMD15_CASQ1)					Modelled age (cal yrs BP)		
**Lab code**	**Depth in core (cm)**	**Material**	**^{14}C age (yrs)**	**^{14}C (+/- yrs)**	**Min.**	**Max.**	**Median**
ULA-6034	117.5	bivalve shell fragment	1570	20	729	1018	894
ULA-5837	176.5	bivalve shell fragment	1850	20	1133	1381	1265
ULA-6035	263.5	bivalve shell fragment	2370	20	1686	1938	1815
ULA-5836	332.5	bivalve shell fragment	2660	25	2122	2332	2234
ULA-6036	341.5	bivalve shell fragment	2705	20	2179	2399	2290
ULA-6037	393.5	bivalve shell fragment	2970	25	2562	2838	2685
ULA-6044	405.5	bivalve shell fragment	3505	20	2674	2978	2806
ULA-6045	460.5	bivalve shell fragment	3505	20	3050	3378	3214
ULA-6046	472.5	bivalve shell fragment	3775	20	3128	3481	3301
ULA-6047	501.5	bivalve shell fragment	3485	25	3274	3700	3456
ULA-5835	543.5	bivalve shell fragment	3745	25	3529	4073	3753
**Lake core (NOW25c)**					**Modelled age (cal yrs BP)**		
**Lab code**	**Depth in core (cm)**	**Material**	**^{14}C age (yrs)**	**^{14}C (+/- yrs)**	**Min.**	**Max.**	**Median**
AAR29941	20	Terrestrial macrofossil	1472	48	1336	1437	1381
AAR29367	40	Terrestrial macrofossil	2122	35	2013	2132	2074
AAR29366	60	Terrestrial macrofossil	2264	38	2282	2396	2353

AAR28578	82	Terrestrial macrofossil	2796	36	2858	3000	2930
AAR29943	100	Terrestrial macrofossil	3297	38	3451	3600	3535
AAR9944	120	Terrestrial macrofossil	3556	34	3781	3913	3839
AAR29945	140	Terrestrial macrofossil	3574	30	3972	4139	4041
AAR29946	148	Terrestrial macrofossil	3744	37	4086	4252	4158
AAR25290	160	Bulk	5525	28	5028	5919	5531
AAR29947	170	Terrestrial macrofossil	5341	33	6099	6346	6263

Supplementary Table 2 – Principal component analysis loadings

**Time-series**	PC1	PC2	PC3
**Marine record**			
Diatoms (excl. *Chaet.* RS)	0.63	0.27	0.73
Chaetoceros RS	0.62	0.40	-0.68
HBI III	-0.47	0.88	0.09
**Lake record**			
$\delta^{15}N$	0.69	0.13	0.02
Sterols	0.41	-0.45	0.71
Cd:Ti	0.12	0.88	0.34
Diatoms	0.58	-0.02	-0.61

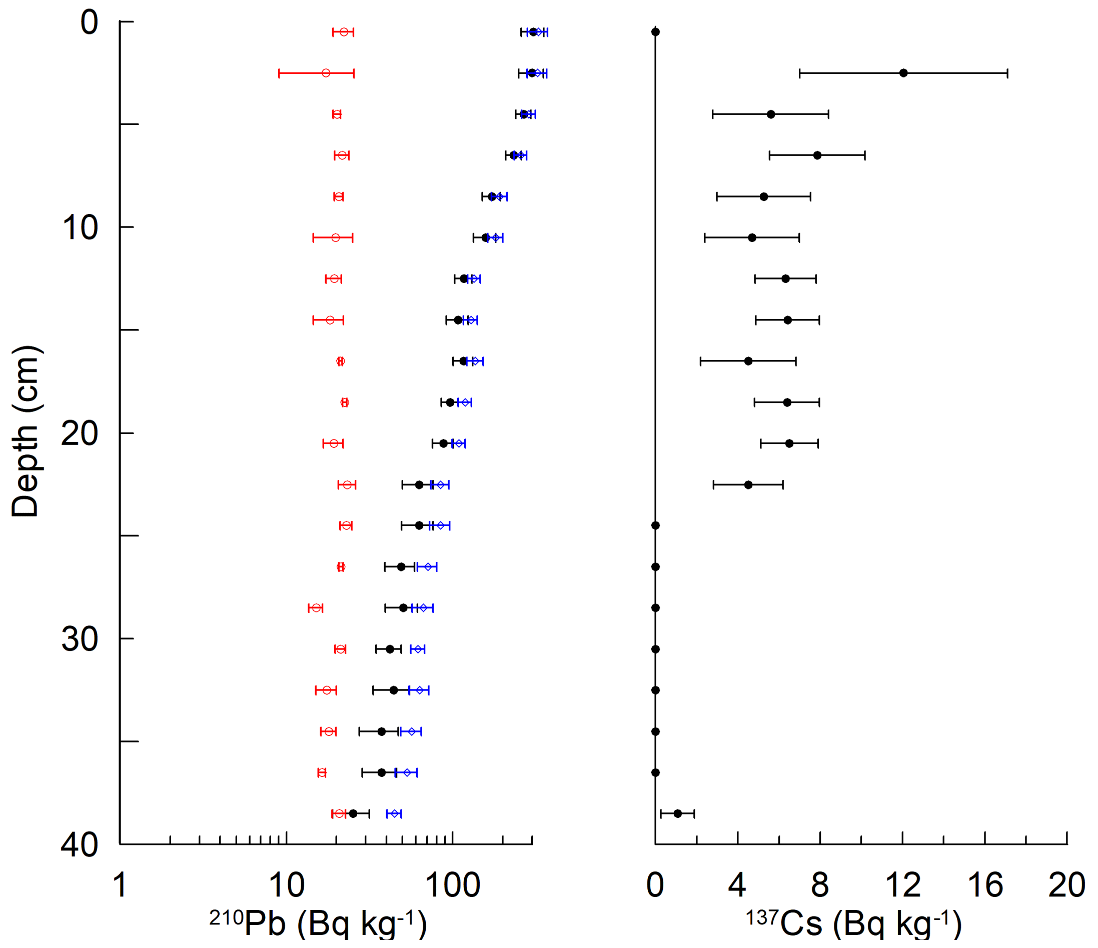

Supplementary Figure 1 – Down-core ^{210}Pb and ^{137}Cs activity for the marine box core record. Open (red) symbols represent supported ^{210}Pb, solid black symbols are unsupported ^{210}Pb, open (blue) diamonds are total ^{210}Pb. Error bars represent one standard deviation.

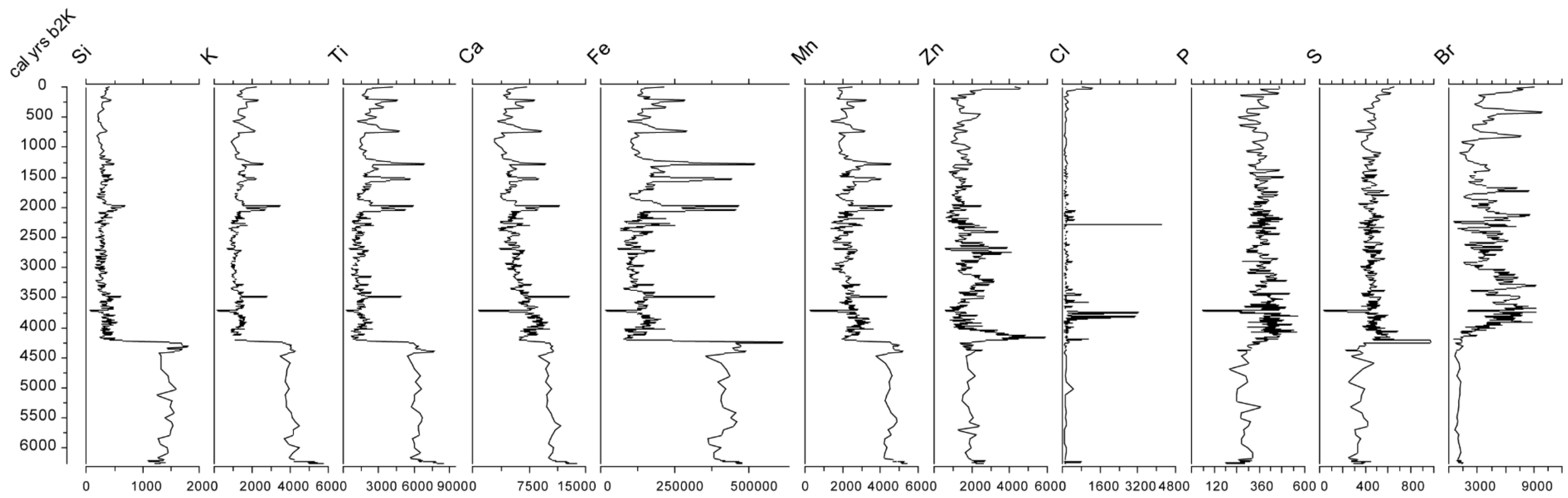

Supplementary Figure 2 – Down-core variation for the major elements detected via X-ray fluorescence (XRF) scanning of the lake core record.

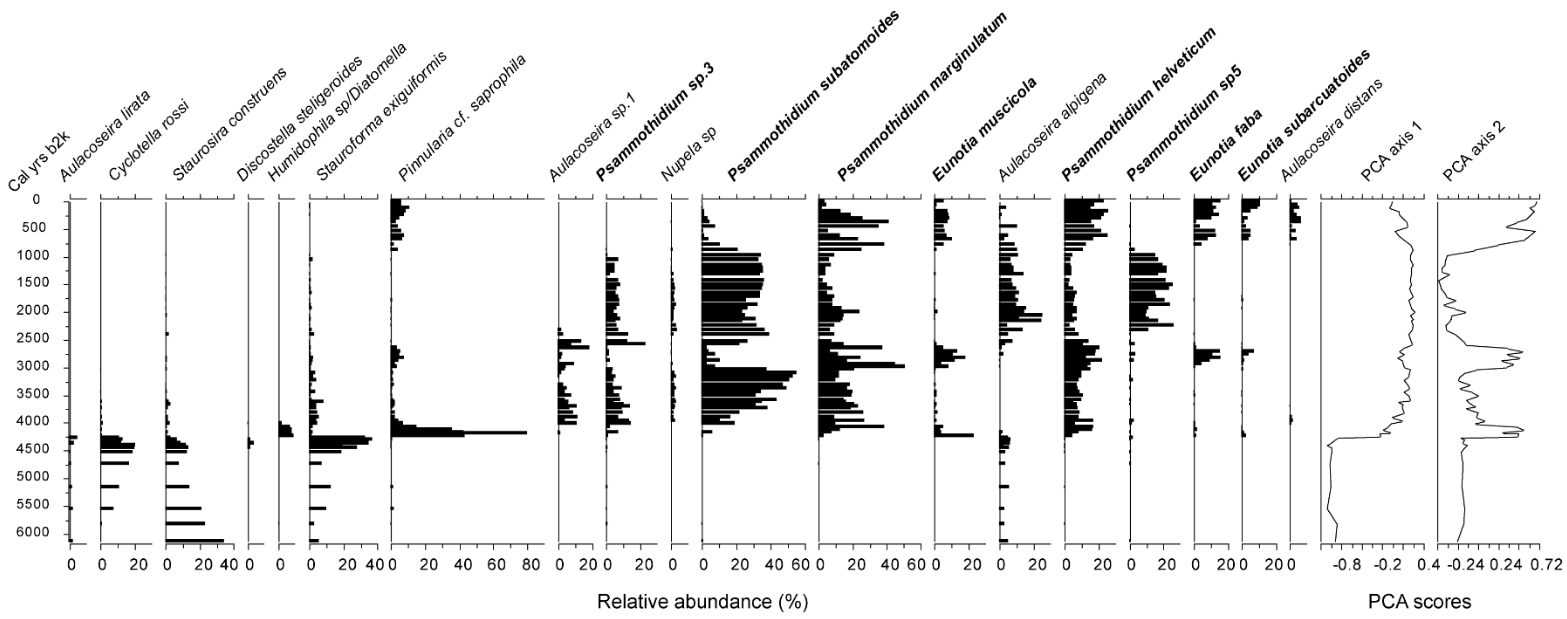

Supplementary Figure 3 – Stratigraphic diagram of the main diatom species present in the lake core record. Species associated with lower pH conditions are marked in bold. Scores for the first and second principal component analyses (PCA) axes are plotted here as shown in Fig. 4. At ca. 4200 cal yrs b2k there is a marked change in the diatom assemblages, summarized by PCA axis 1 and indicating a decrease in lake pH, whereas PCA axis 2 captures the variability between slightly (*P. subatomoides*) and moderately to strongly acidophilous species (*P. marginulatum*, *P. helveticum*, *Eunotia* species).

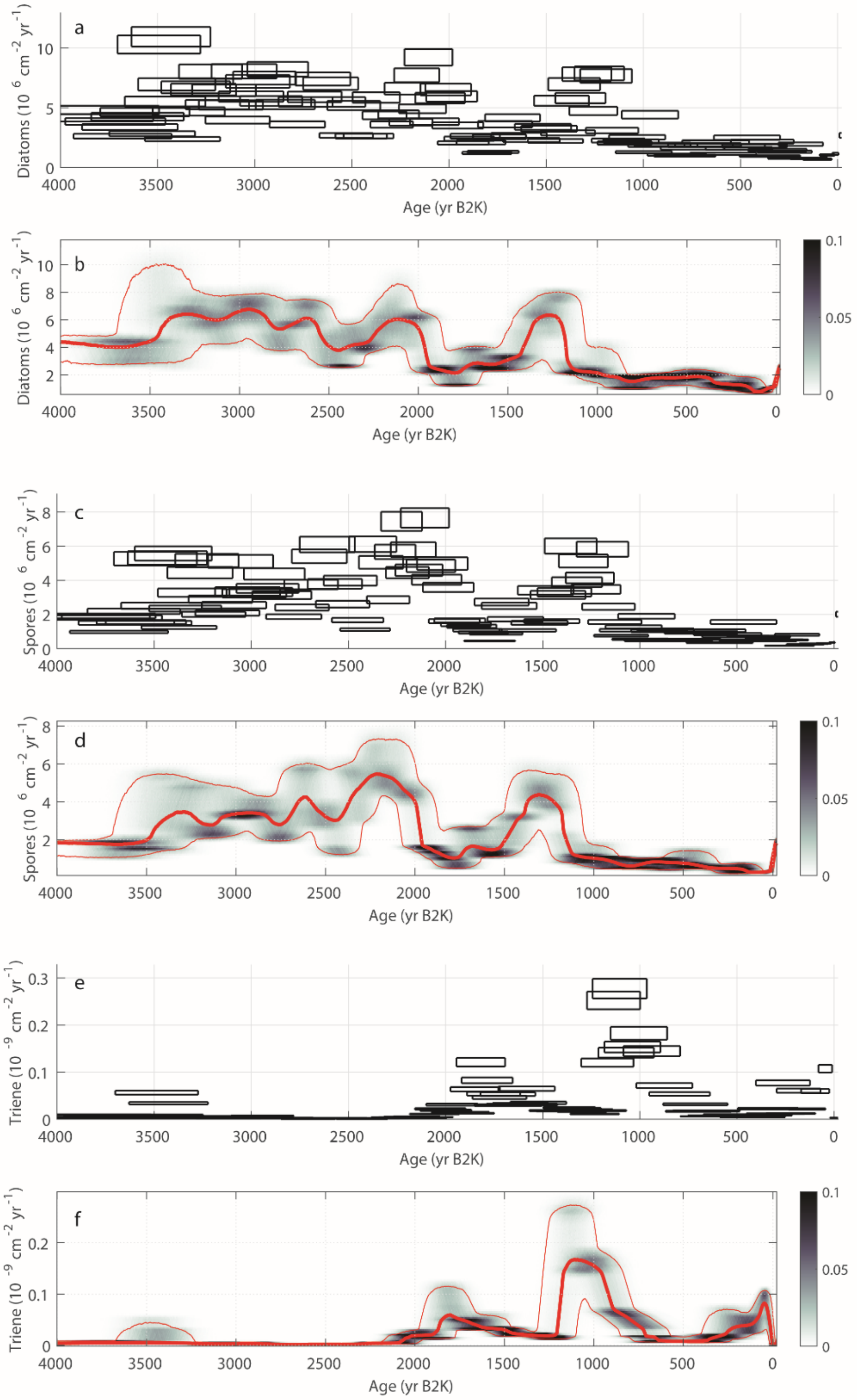

Supplementary Figure 4 – Monte Carlo simulations used to derive principal components for the marine record. a, c, e: Boxplots defining conservative uncertainty intervals in both age estimation (X) and measured values (Y) used for the 10, 000 Monte Carlo simulations. b, d, f: 2D frequency histograms depicting the probability density of a given measurement at a given time for each constituent time series (Diatoms, *Chaetoceros* spores and Triene) resulting from 10,000 Monte Carlo simulations.

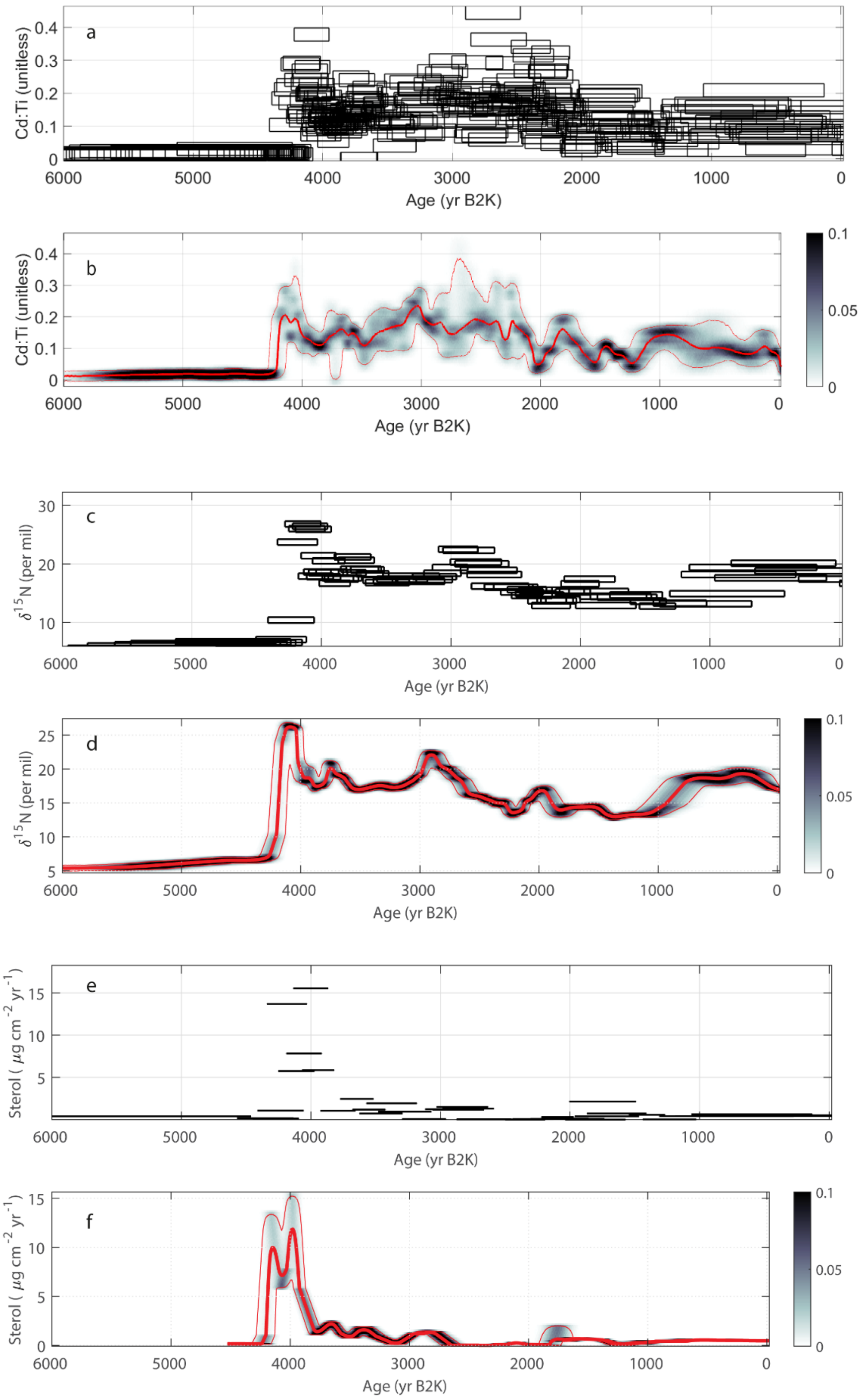

a
Cd:Ti (unitless)
Age (yr B2K)
b
Cd:Ti (unitless)
Age (yr B2K)
c
δ15N (per mil)
Age (yr B2K)
d
δ15N (per mil)
Age (yr B2K)
e
Sterol (μg cm -2 yr -1)
Age (yr B2K)
f
Sterol (μg cm -2 yr -1)
Age (yr B2K)

Supplementary Figure 5 – Monte Carlo simulations used to derive principal components for the lake proxy records Cd:Ti, $\delta^{15}N$ and sterols. a, c, e: Boxplots defining conservative uncertainty intervals in both age estimation (X) and measured values (Y) used for the 10, 000 Monte Carlo simulations. b, d, f: 2D frequency histograms depicting the probability density of a given measurement at a given time for each constituent time series resulting from 10,000 Monte Carlo simulations.

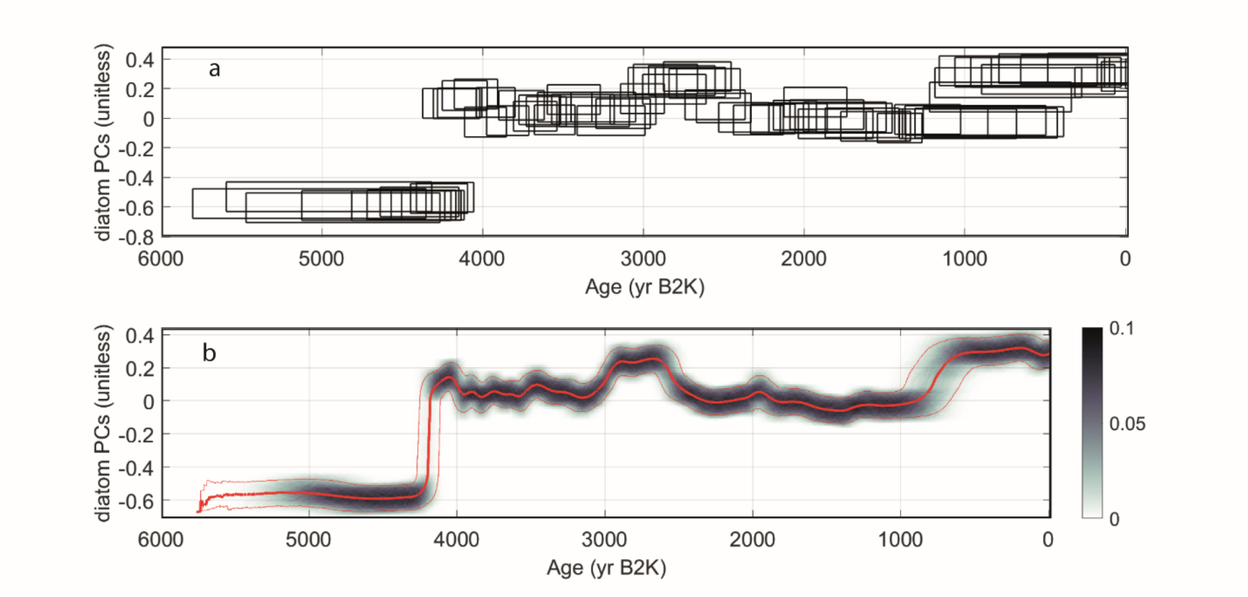

Supplementary Figure 6 – Monte Carlo simulations used to derive principal components for the lake diatom assemblages. a: Boxplots defining conservative uncertainty intervals in both age estimation (X) and measured values (Y) used for the 10, 000 Monte Carlo simulations. b: 2D frequency histograms depicting the probability density of the main PC axes at a given time resulting from 10,000 Monte Carlo simulations.

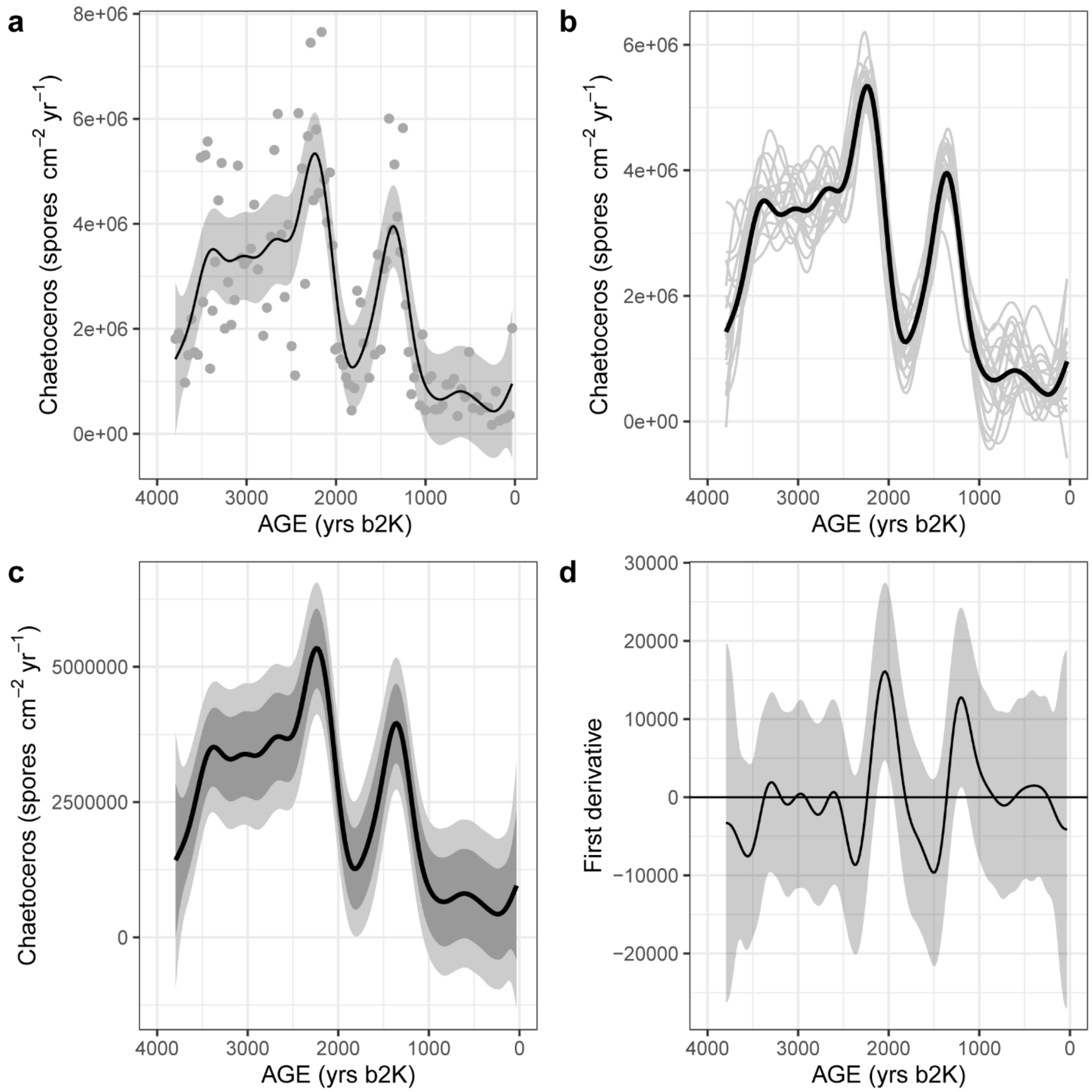

Supplementary Figure 7 – Generalised additive model results for the marine proxy "*Chaetoceros* spores". a) fitted model and 95% confidence interval; b) 20 random draws of the posterior distribution of the model and their mean, which summarise the uncertainty; c) confidence intervals derived from the random draws of the posterior distribution and d) plot of the first derivative and its confidence interval used to identify periods of significant change, where the 95% confidence interval does not cover zero (marked as stars in Figure 4). For further details see methods.

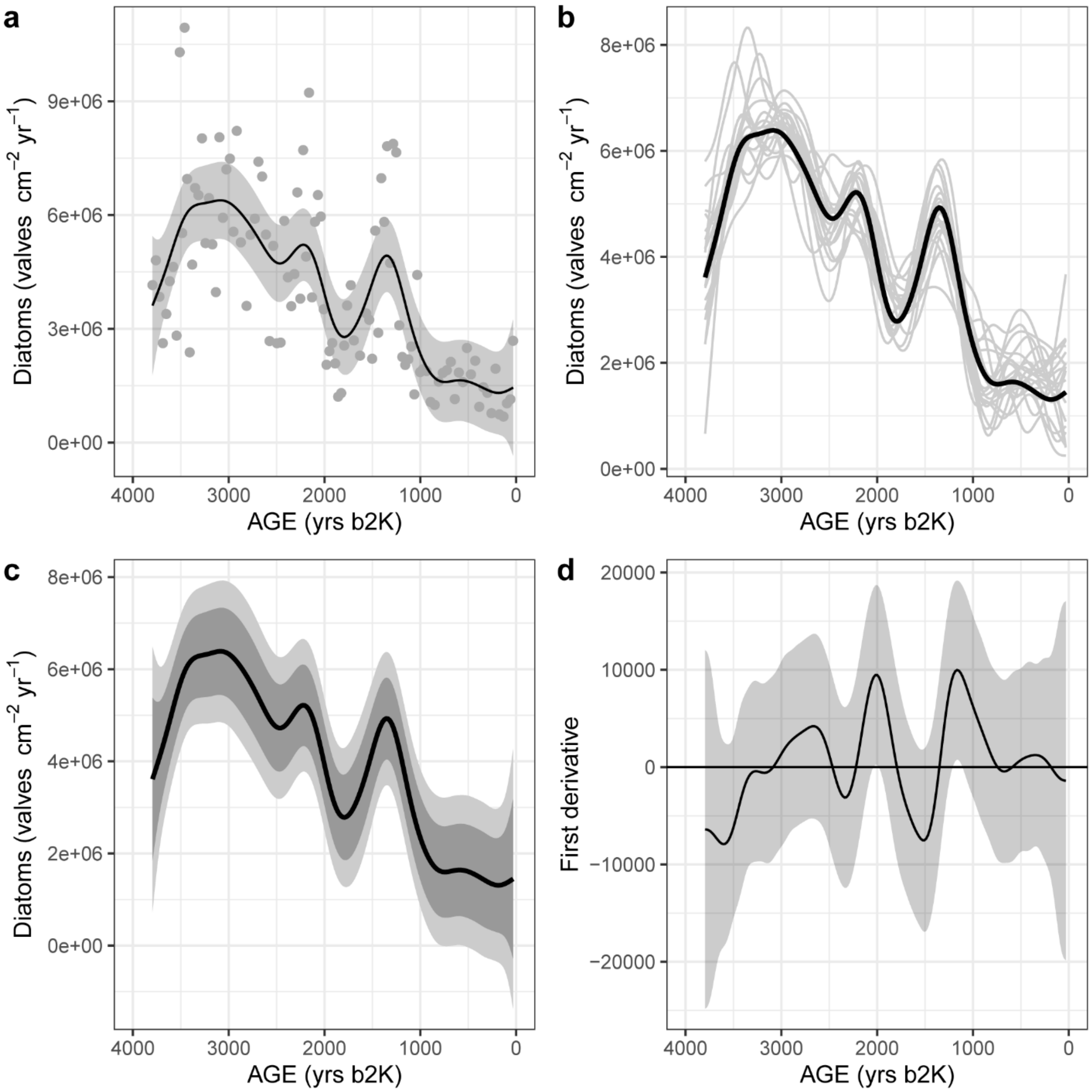

Supplementary Figure 8 - Generalised additive model results for the marine proxy "Diatoms". a) fitted model and 95% confidence interval; b) 20 random draws of the posterior distribution of the model and their mean, which summarise the uncertainty; c) confidence intervals derived from the random draws of the posterior distribution and d) plot of the first derivative and its confidence interval, used to identify periods of significant change, where the 95% confidence interval does not cover zero (marked as stars in Figure 4). For further details see methods.

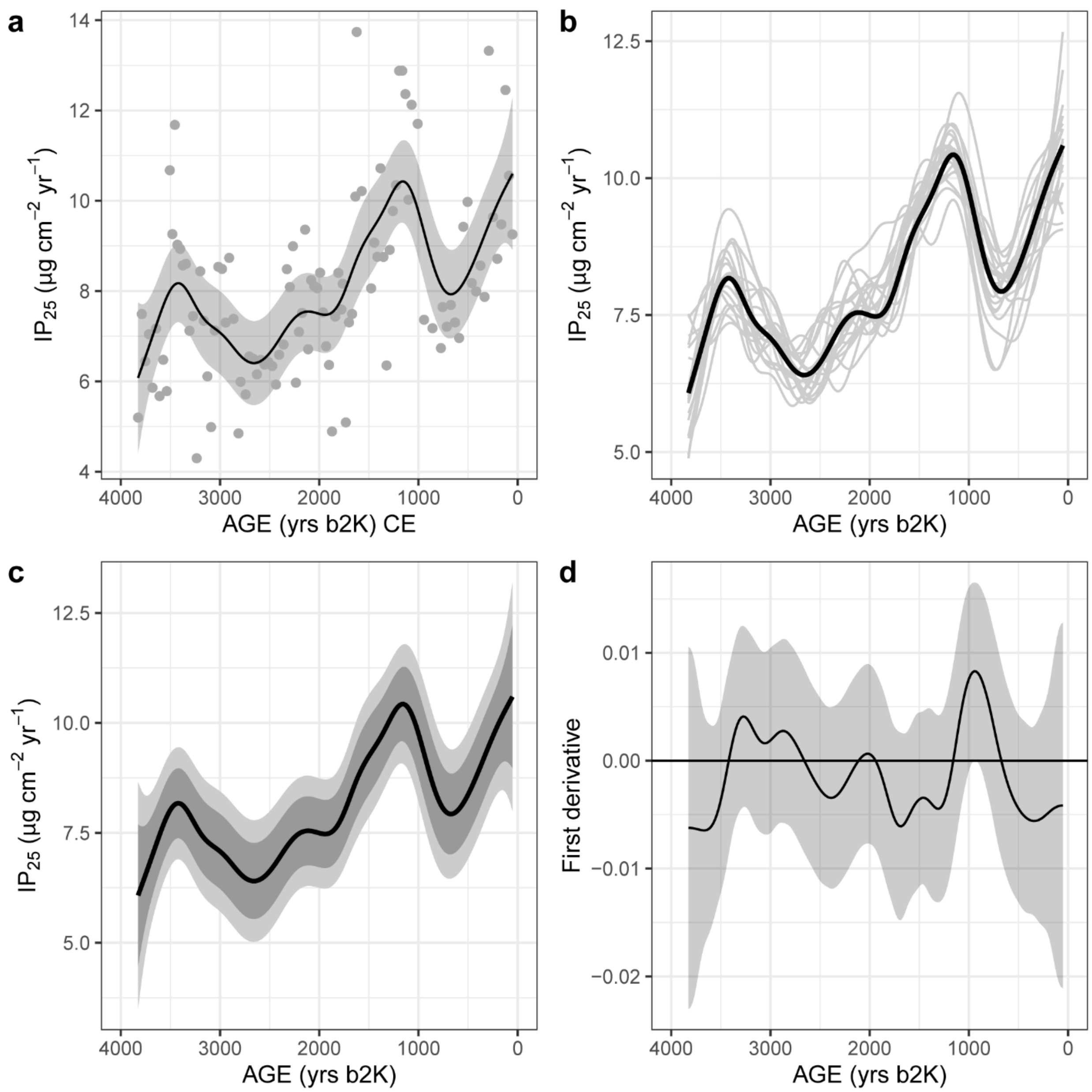

Supplementary Figure 9 - Generalised additive model results for the marine proxy "IP_{25}". a) fitted model and 95% confidence interval; b) 20 random draws of the posterior distribution of the model and their mean, which summarise the uncertainty; c) confidence intervals derived from the random draws of the posterior distribution and d) plot of the first derivative and its confidence interval, used to identify periods of significant change, where the 95% confidence interval does not cover zero (marked as stars in Figure 4). For further details see methods.

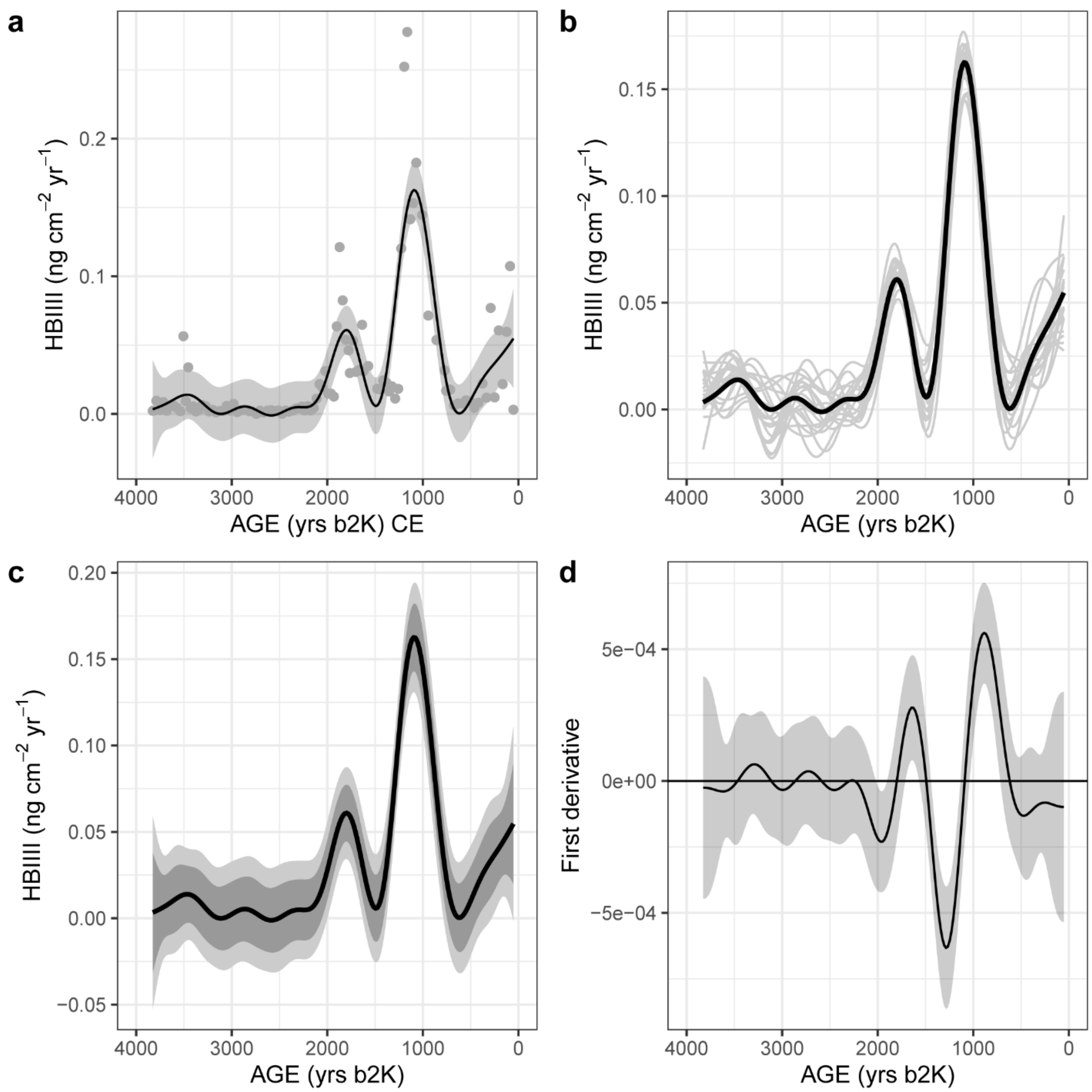

Supplementary Figure 10 - Generalised additive model results for the marine proxy "HBI III (Triene)" a) fitted model and 95% confidence interval; b) 20 random draws of the posterior distribution of the model and their mean, which summarise the uncertainty; c) confidence intervals derived from the random draws of the posterior distribution and d) plot of the first derivative and its confidence interval, used to identify periods of significant change, where the 95% confidence interval does not cover zero (marked as stars in Figure 4). For further details see methods.

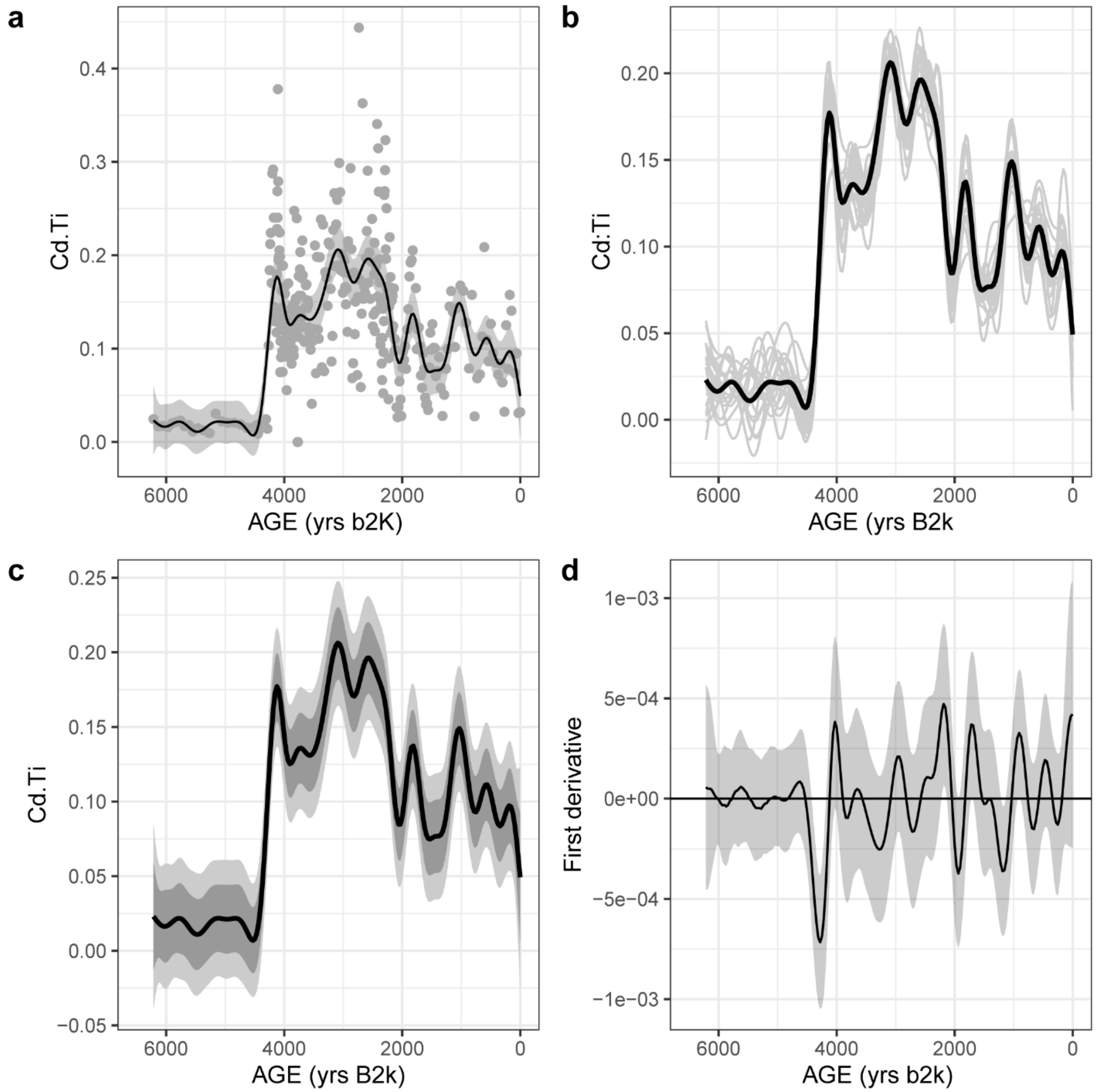

Supplementary Figure 11 - Generalised additive model results for the lake indicator of Little Auk influence "Cd:Ti" a) fitted model and 95% confidence interval; b) 20 random draws of the posterior distribution of the model and their mean, which summarise the uncertainty; c) confidence intervals derived from the random draws of the posterior distribution and d) plot of the first derivative and its confidence interval, used to identify periods of significant change, where the 95% confidence interval does not cover zero (marked as stars in Figure 4). For further details see methods.

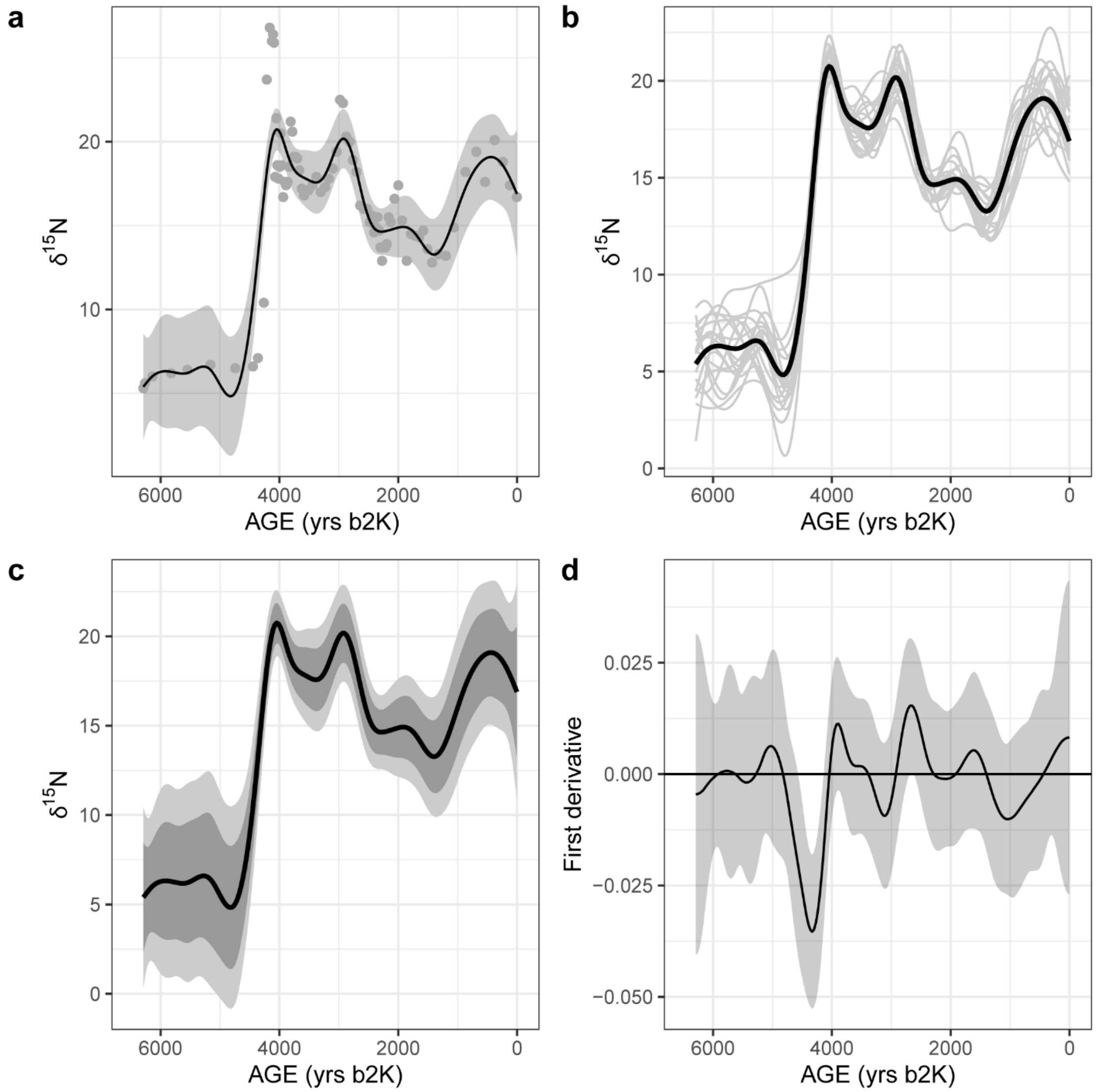

Supplementary Figure 12 - Generalised additive model results for the lake indicator of little auk influence "$\delta^{15}N$" a) fitted model and 95% confidence interval; b) 20 random draws of the posterior distribution of the model and their mean, which summarise the uncertainty; c) confidence intervals derived from the random draws of the posterior distribution and d) plot of the first derivative and its confidence interval, used to identify periods of significant change, where the 95% confidence interval does not cover zero (marked as stars in Figure 4). For further details see methods.

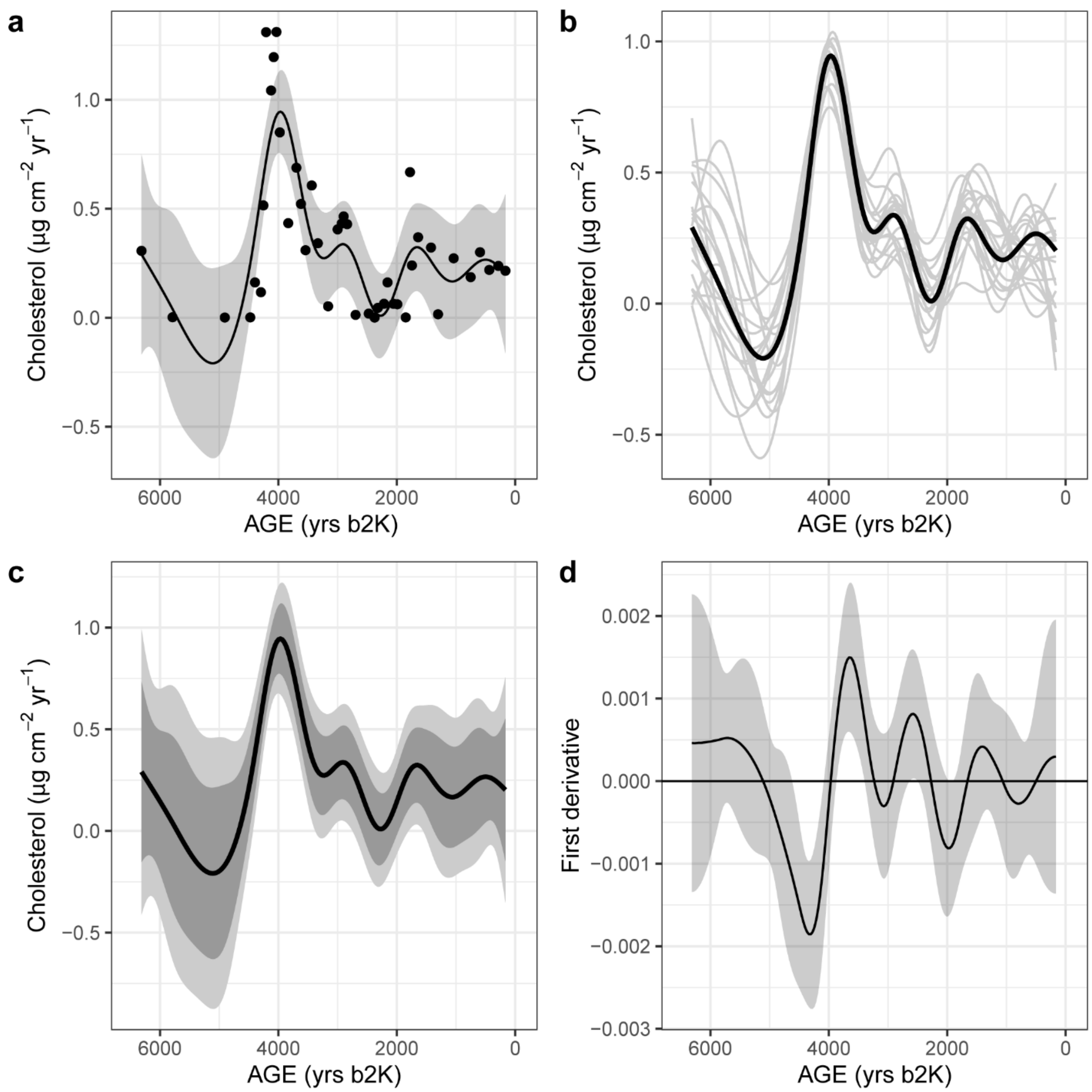

Supplementary Figure 13 - Generalised additive model results for the lake indicator of Little Auk influence "Cholesterol" a) fitted model and 95% confidence interval; b) 20 random draws of the posterior distribution of the model and their mean, which summarise the uncertainty; c) confidence intervals derived from the random draws of the posterior distribution and d) plot of the first derivative and its confidence interval, used to identify periods of significant change, where the 95% confidence interval does not cover zero (marked as stars in Figure 4). For further details see methods.

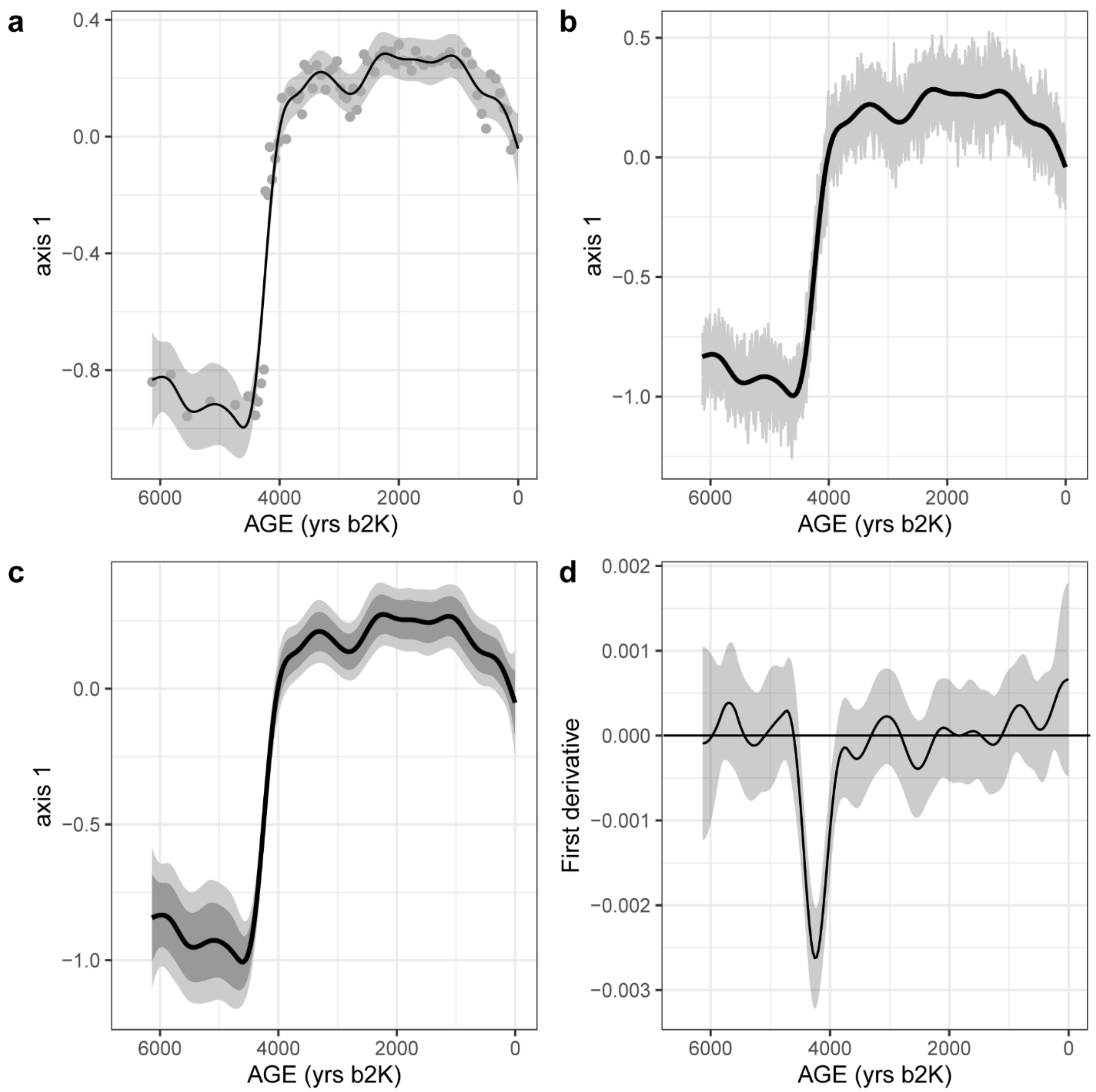

Supplementary Figure 14 - Generalised additive model results for the first PCA axis - lake diatom assemblages a) fitted model and 95% confidence interval; b) 20 random draws of the posterior distribution of the model and their mean, which summarise the uncertainty; c) confidence intervals derived from the random draws of the posterior distribution and d) plot of the first derivative and its confidence interval, used to identify periods of significant change, where the 95% confidence interval does not cover zero (marked as stars in Figure 4). For further details see methods.

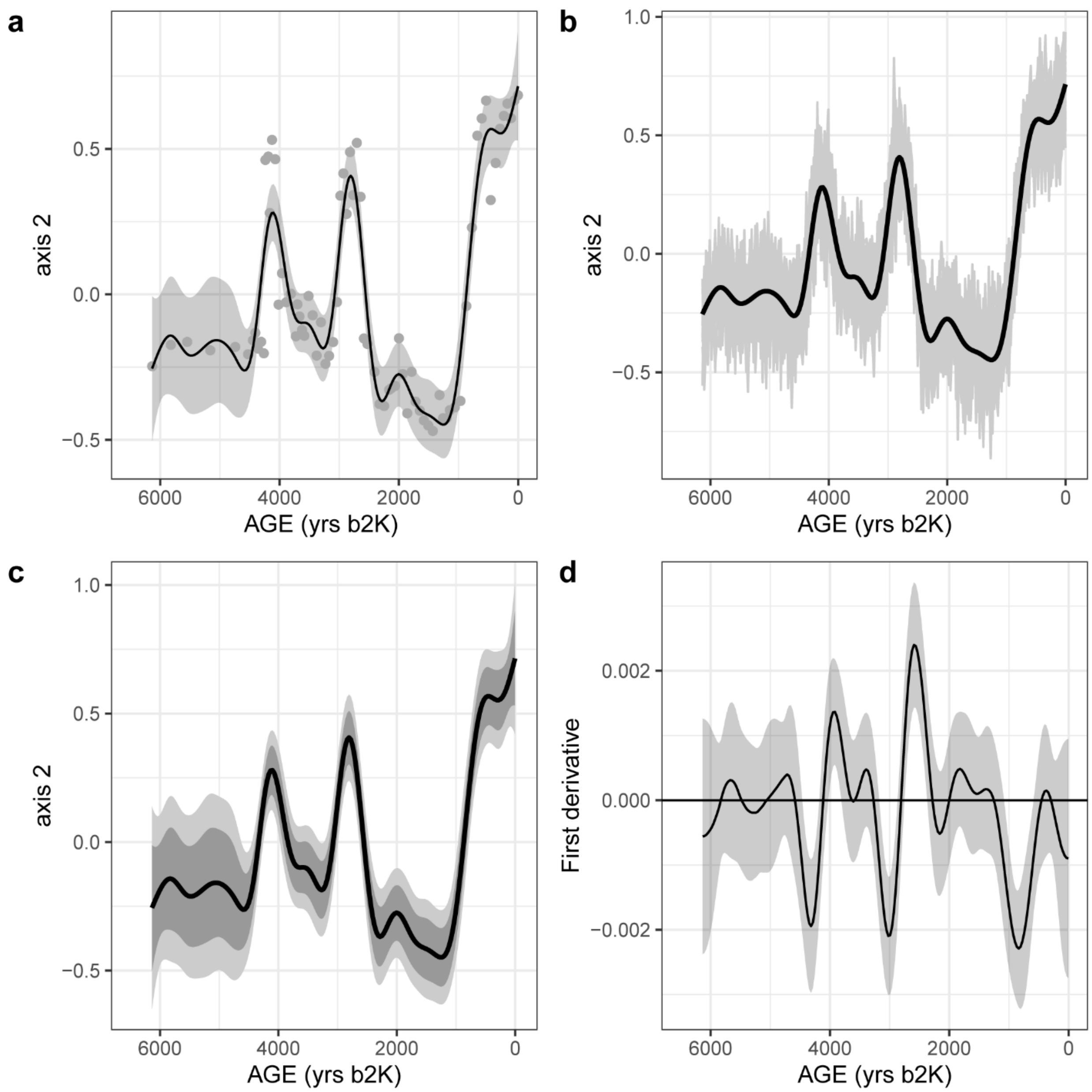

Supplementary Figure 15 - Generalised additive model results for the second PCA axis - lake diatom assemblages a) fitted model and 95% confidence interval; b) 20 random draws of the posterior distribution of the model and their mean, which summarise the uncertainty; c) confidence intervals derived from the random draws of the posterior distribution and d) plot of the first derivative and its confidence interval, used to identify periods of significant change, where the 95% confidence interval does not cover zero (marked as stars in Figure 4). For further details see methods.

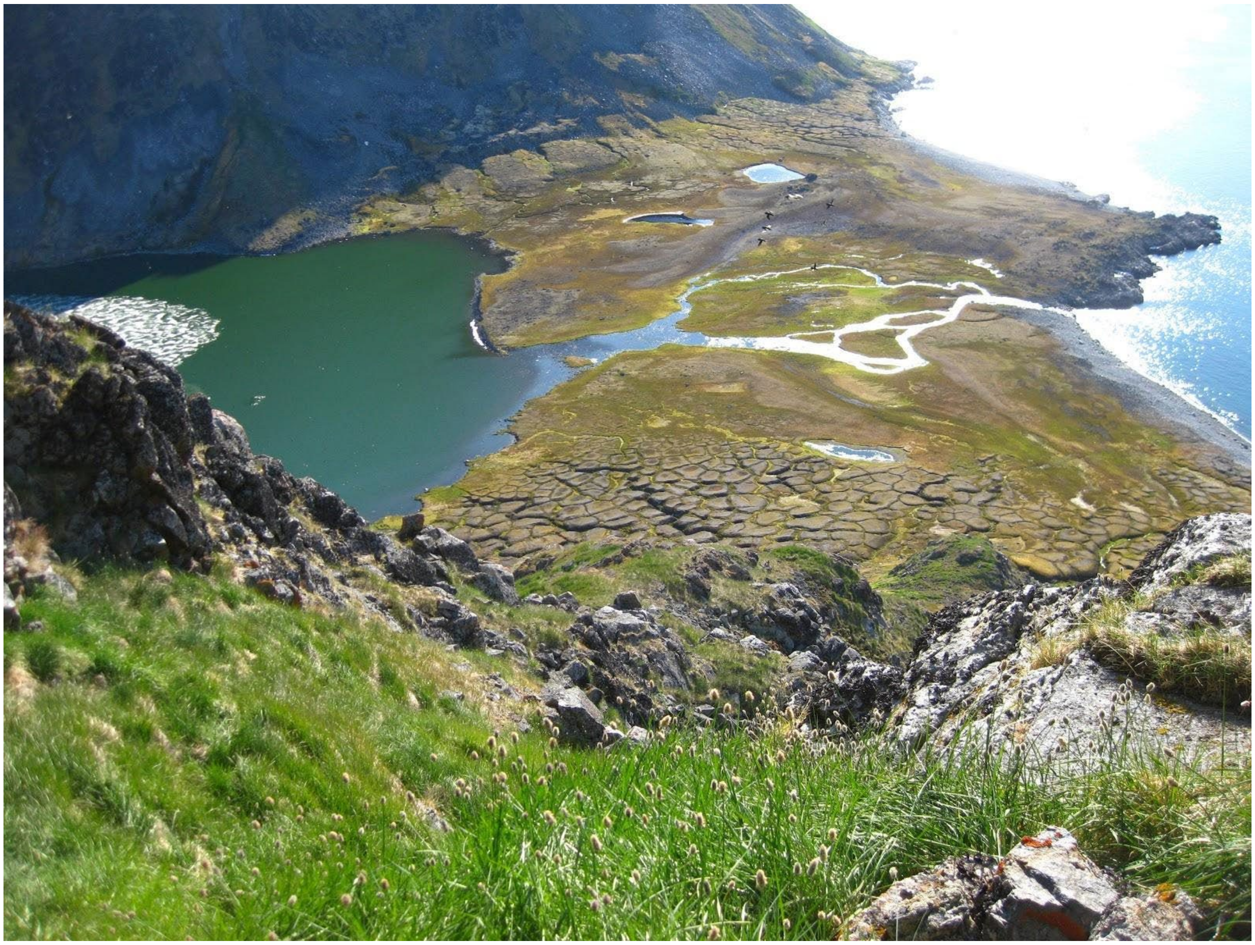

Supplementary Figure 16 - Aerial view of the lake catchment showing extensive peat formation and lush catchment vegetation.
